# Space-time migration patterns and risk of HIV acquisition in rural South Africa

**DOI:** 10.1097/QAD.0000000000001292

**Published:** 2016-12-02

**Authors:** Adrian Dobra, Till Bärnighausen, Alain Vandormael, Frank Tanser

**Affiliations:** a Department of Statistics, Department of Biobehavioral Nursing and Health Systems, Center for Statistics and the Social Sciences, and Center for Studies in Demography and Ecology, University of Washington, Seattle, Washington; b Africa Health Research Institute, University of KwaZulu-Natal, Mtubatuba, South Africa; Institute for Public Health, Faculty of Medicine, University of Heidelberg, Heidelberg, Germany; and Department of Global Health and Population, Harvard T.H. Chan School of Public Health, Boston, Massachusetts, USA; c Africa Health Research Institute, University of KwaZulu-Natal, Mtubatuba, South Africa; d Africa Health Research Institute, School of Nursing and Public Health, University of KwaZulu-Natal, Durban; Centre for the AIDS Programme of Research in South Africa – CAPRISA, University of KwaZulu-Natal, Congella, South Africa.

**Keywords:** HIV acquisition, migration, mobility, South Africa, sub-Saharan Africa

## Abstract

**Objective:**

To quantify the space-time dimensions of human mobility in relationship to the risk of HIV acquisition.

**Methods:**

We used data from the population cohort located in a high HIV prevalence, rural population in KwaZulu-Natal, South Africa (2000–2014). We geolocated 8006 migration events (representing 1 028 782 km traveled) for 17 743 individuals (≥15 years of age) who were HIV negative at baseline and followed up these individuals for HIV acquisition (70 395 person-years). Based on the complete geolocated residential history of every individual in this cohort, we constructed two detailed time-varying migration indices. We then used interval-censored Cox proportional hazards models to quantify the relationship between the migration indices and the risk of HIV acquisition.

**Results:**

In total, 17.4% of participants migrated at least once outside the rural study community during the period of observation (median migration distance = 107.1 km, interquartile range 18.9–387.5). The two migration indices were highly predictive of hazard of HIV acquisition (*P* < 0.01) in both men and women. Holding other factors equal, the risk of acquiring HIV infection increased by 50% for migration distances of 40 km (men) and 109 km (women). HIV acquisition risk also increased by 50% when participants spent 44% (men) and 90% (women) of their respective time outside the rural study community.

**Conclusion:**

This in-depth analysis of a population cohort in a rural sub-Saharan African population has revealed a clear nonlinear relationship between distance migrated and HIV acquisition. Our findings show that even relatively short-distance migration events confer substantial additional risk of acquisition.

## Introduction

Despite the availability of effective interventions, the rate of new HIV infections remains unacceptably high in sub-Saharan Africa [1]. Population mobility has long been recognized as one of the main catalysts of the spread of the HIV epidemic [2–10]. HIV is transmitted through sexual networks with heterogeneous spatial and sociodemographic structure. Mobile individuals that periodically change their residences between urban, rural, and peri-urban areas, disconnect from the local sexual network associated with the origin of their move, and are likely to connect with another local sexual network associated with their destination. Even if mobile individuals continue to maintain ties with the partners they leave behind, their sexual behavioral patterns are fundamentally different from those of less mobile individuals that travel only for shorter periods of time and over shorter distances because of uprooting, losing social ties, and needing to find new social networks. Furthermore, they experience a higher likelihood of becoming a victim of violence, including sexual violence [2–6,9,11–25].

Although links between migration and HIV have been established for some time [2,10,11,16,26–29], there have been no longitudinal studies conducted to clearly understand the relationship between different space-time patterns of migration and the prospective risk of HIV acquisition. Cross-sectional studies that link different patterns of migration with higher levels of HIV prevalence cannot differentiate between migration episodes that preceded or followed HIV acquisition: the former episodes could have contributed to changes in sexual behavior that led to HIV acquisition, whereas the latter episodes could have resulted from individuals seeking family support, healthcare, or from their desire to avoid social stigma [2,20]. Yet, such knowledge is of critical importance in the design and implementation of effective intervention packages.

In this article, we analyze comprehensive space-time human mobility patterns in a rural area in northern KwaZulu-Natal, South Africa based on detailed longitudinal sociodemographic and individual HIV surveillance data available from the Africa Health Research Institute. Our use of comprehensive longitudinal information on HIV surveillance, sexual behavior, and sociodemographic data, coupled with complete geolocated residential histories, give us the ability to ascertain causal relationships between differing mobility patterns and subsequent risk of HIV acquisition. To the best of our knowledge, this study is the first to employ complete geolocated residential histories to quantify the space-time dimensions of human mobility in relationship to the risk of HIV acquisition.

## Methods

### Setting and data source

This is a population-based cohort study based on data collected between 2004 and 2014 from the demographic information system of the Africa Health Research Institute located within the Umkhanyakude district of northern KwaZulu-Natal, South Africa [30]. This community is characterized by frequent migration (38% of men and 32% of women were nonresident in 2008) [31], low marital rates (only 23% of men and 31% of women have ever been married) [32], late marriage especially for men, polygamous marriages (about 14% of all marriages for men and 12% of all marriages for women) [32] and multiple sexual partnerships, as well as by poor knowledge and disclosure of HIV status [33].

The Africa Health Research Institute collects data on the characteristics of households and individuals who belong to family units in the rural study community. Births, deaths, and migrations are recorded every 4 months, whereas measures of socioeconomic status are recorded annually. The Africa Health Research Institute also conducts annual population-based HIV surveillance and sexual behavior surveys for all consenting individuals aged 15 or older. Field workers obtain blood by finger prick from each consenting individual in this open cohort; more than 80% of individuals contacted agree to be tested at least once. Individuals become part of the HIV cohort when they turn 15 years old, or when they in-migrate into the rural study community. Sexual behavior data are collected by face-to-face interviews [30].

The demographic information system collects data about all the individuals that are members of a family unit or a household in the rural study community irrespective of the current residency status. A migration event is defined as a change in residency [30]. Fieldworkers record the origin place of residence, the destination place of residence, and the date of the move for every migration event at the time of their visit. In-migrations are migration events in which the origin is outside the rural study community, and the destination is inside the rural study community. Out-migrations are migration events in which the origin is inside the rural study community, and the destination is outside that community. External migration events comprise in-migrations, out-migrations, and also those migration events whose origin and destination are both located outside the rural study community.

### Cohort description

From the entire resident population under surveillance at the Africa Health Research Institute, we focus on those individuals that had at least two recorded HIV tests after turning 15 years old, and whose first test was negative. We require these repeat-testers to have been resident members of at least one household in the surveillance area between 1 January 2004 and 31 December 2014. In our analysis, the exposure period for a repeat-tester starts at the time of their first HIV test, and ends at the time of their first HIV positive test for seroconverters, or at the time of their last HIV negative test for those that did not seroconvert.

As opposed to other studies focusing on migration and HIV [4,9–11,23–25] (see also Table S1 in the Supporting Information), we refrain from classifying repeat-testers as migrants or nonmigrants because this dichotomization cannot capture complex, heterogeneous patterns of repeated movement inside and outside the rural study community. Instead, we adopt a representation that is more fluid: repeat-testers can have zero, one, two, or more internal and external migration events that last shorter or longer periods of time over the course of their lifetime. We divided the exposure period of a repeat-tester into nonoverlapping exposure episodes based on changes of residence, age, marital status, changes in educational status, and new HIV tests. Inside the rural study community, an individual resides in homesteads whose geolocation is measured with an accuracy of less than 2 m. When an individual resides outside the rural study community, the geolocation of their place of residence is determined based on place names collected by field workers during interviews of family members in the repeat-tester's household, as described below.

### Geocoding places of residency outside the rural study community

Each description of a place of residency was geocoded using three online services: HERE Geocoder (https://here.com/en), Bing Maps REST Services, and Google Maps Geocoding. These three services allow the determination of latitude and longitude coordinates of a location specified by a text address by matching it against their internal databases of known addresses. The use of three geocoding services provides robustness of the coordinates identified because HERE, Bing, and Google employ various string matching rules and techniques. The latitude and longitude coordinates that were closest to the rural study community were chosen for the queries for which multiple latitude and longitude coordinates were available. The queries that were successfully geolocated by a single API were manually checked, and included in the database on a case-by-case basis after assessing the validity of the original query. For details, see Section S4 in the Supporting Information.

### Construction of migration indices

We constructed two time-varying measures of migration that quantify key spatiotemporal features of migration patterns of repeat-testers. Our first migration index, which we term ‘time outside,’ represents the proportion of time associated with periods of residence outside the rural study community of a repeat-tester. Time outside is exclusively a temporal measure that is independent of the geolocation of the residencies occupied by a repeat-tester. Geolocation data are, however, essential in the construction of our second migration index. This measure, which we call ‘migration distance,’ represents the sum of the distances between consecutive residences occupied by a repeat-tester in a given year. Additional explanations related to construction of the migration indices are provided in Section S2 in the Supporting Information.

### Statistical analysis

Separately for men and women, we employed Cox proportional hazards models of time to HIV seroconversion to investigate the effect of the spatiotemporal migration patterns of repeat-testers on their hazard of HIV acquisition. For each time-dependent migration index, we fitted a model adjusted for well established individual-level determinants of HIV incidence in this population (reporting more than one partner in the previous 12 months over the duration of the study, marital status, number of years of education, perceived financial status, and age) [9,34,35].

We followed up 20 989 repeat-testers during the study period (2000–2014) that met our inclusion criteria. As there was no requirement for the Africa Health Research Institute Demographic Surveillance System survey participants to answer all the questions they were asked, some data about age, marital status, number of years of education, perceived financial status, or sexual history were missing. In total, 8155 (99.4%) men and 12 692 (99.3%) women in our incidence cohort had complete information for these risk factors for all their exposure episodes. We dropped those repeat-testers for which we could not determine the geolocation of their places of residence outside the rural study community. Geolocation data availability did not vary significantly by sex: 6995 (85.8%) of men and 10 749 (84.7%) of women with complete sociodemographic and sexual history data also had complete geolocated residential histories.

In the resulting data, the median time elapsed between the date of the last HIV-negative test and the date of the first HIV-positive test for seroconverters were 2.86 [interquartile range (IQR) 3.48] years for men and 2.12 (IQR 2.82) years for women (Tables S2 and S3 in the Supporting Information). The times to HIV seroconversion are therefore interval censored. We used a method recently developed for fitting Cox proportional hazards model with interval censoring and time-dependent covariates to fully capture the effects of uncertainty around the exact date of HIV seroconversion [36]. Under this approach which allows for an arbitrary number of testing times for each repeat-tester, the cumulative hazard function of the seroconversion time of a repeat-tester is defined conditional on a linear combination of time-dependent and time-independent covariates that are specific to that individual, and an unknown cumulative baseline hazard function that remains constant across individuals. The vector of regression parameters associated with the covariates and the cumulative baseline hazard function are estimated with an expectation-maximization algorithm with good convergence properties even for large sample sizes. All analyses were done in R version 3.2.3 ‘Wooden Christmas-Tree’ [37].

## Results

Over the duration of the study (2000–2014), the crude HIV incidence rate for men was 2.16 cases [95% confidence interval (CI) 1.98–2.34] per 100 person-years (547 new infections in 25 284.57 person-years of follow-up). For women, the HIV incidence rate was 3.27 cases (95% CI 3.11–3.44) per 100 person-years (1476 new infections in 45 110.17 person-years of follow-up). Table S4 in the Supporting Information shows crude HIV incidence rates broken down by 5-year age groups, and by calendar year. The highest incidence rate for men was measured in the 25–29 age group: 5.79 (95% CI 4.79–6.80) per 100 person-years. In women, the highest incidence rate was measured in the 20–24 age group: 9.11 (95% CI 8.40–9.82) per 100 person-years, whereas in the 25–29 group the incidence rate is 7.03 (95% CI 6.07–7.99) per 100 person-years.

**Fig. 1:**
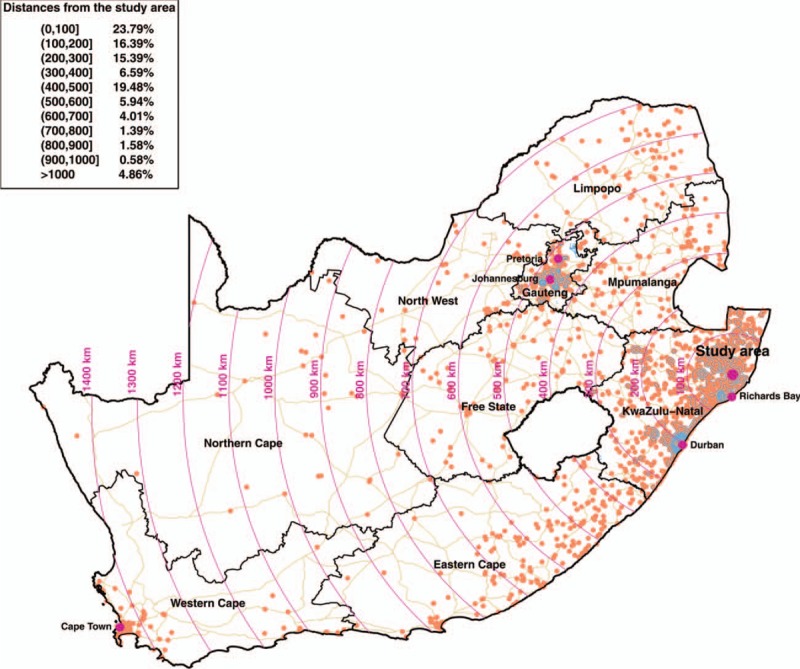
Map of the spatial distribution of the migration locations in South Africa (orange dots).

The geolocations of 8006 migration events (representing 1 028 782 km traveled) for all 17 743 individuals (≥15 years of age) in the incidence cohort are mapped in Fig. 1. Migration events cluster around the metropolitan areas of Richards Bay, Durban, Johannesburg, and Pretoria. These findings are consistent with an earlier study [28] focusing on labor circular migration in the rural study community. Figures S1 and S2 in the Supporting Information depict the space-time characteristics of migration events outside the rural study community. Overall, the spatiotemporal external migration patterns of men and women are strikingly similar. The distances traveled during most migration events are about the length of a round trip from the rural study community to Richards Bay (110 km), Durban (409 km), Johannesburg/Pretoria (945 km). The duration of the majority of the periods resided outside the study area is less than 2 years (median 16.0 months, IQR 8.0–29.5 months).

The location of the Africa Health Research Institute rural study community is represented by a larger violet dot, whereas cities that are key migration destinations are represented by smaller violet dots. We mapped the motorways, trunk, and primary roads in South Africa, together with the boundaries of the nine South African provinces.

In this cohort, we observed at least one external migration episode in 17.4% participants during the period of observation (median observation time 55.8 months, IQR 31.9–84.0 months). The median distance migrated among those that moved outside the rural study community was 107.1 km (IQR 18.9–387.5 km), whereas the median proportion of time resided outside the rural study community was 30.8% (IQR 14.4–55.7%). Table 1 presents descriptive characteristics of the frequency of external migration episodes. The external migration rates for men are approximately 23% in the 20–24 and 25–29 age groups, whereas for women they reach 26% in the 20–24 age group, and 19% in the 25–29 age group. In men, these rates drop below 10% only in the above 45-years-old age group. Women move outside the rural study community a lot less after the age of 30.

**Fig. 2:**
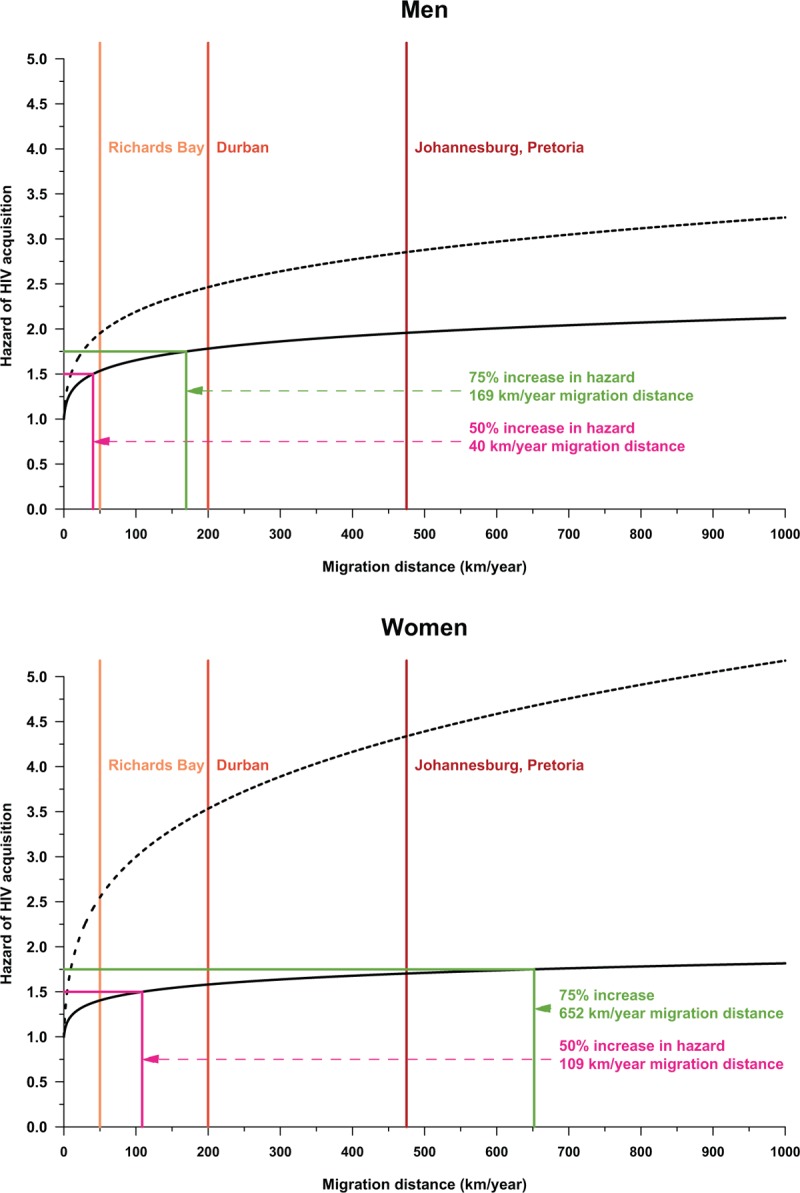
Relationship between the hazard of HIV acquisition and migration distance expressed in kilometers per year for men (top) and women (bottom).

Table 2 summarizes the estimates of adjusted hazard ratios associated with our migration indices from the interval-censored Cox proportional hazards models of time to HIV seroconversion. Both migration indices were significantly associated with risk of acquisition of infection in men and women (*P* < 0.01). When we plot these hazard ratios, we see a clear nonlinear relationship between distance migrated and risk on acquisition of infection (Fig. 2). The relationship is characterized by an initial rapid increase in risk of infection within the first 50–100 km followed by an attenuation in the rate of increase in risk thereafter. The graph shows that men who relocate as close as Richards Bay (55 km away) increase their hazard of acquiring HIV by 50% (compared with men that continue to live inside the rural study community). The risk of acquisition of infection increases to 75% for those men who migrate the distance to Durban (205 km away). The distance–HIV risk relationship was also clearly evident for women: those who migrate the distance to Durban increased their hazard of acquiring HIV by 50%, whereas women who moved to Johannesburg or Pretoria (473 km away) increased their hazard of acquiring HIV by approximately 75%.

The black solid lines give the adjusted hazard of HIV acquisition for migration distance, whereas the dotted lines give the unadjusted hazard of HIV acquisition. The colored lines show the number of kilometers per year of migration distance that correspond with a 50% or a 75% increase in the adjusted hazard of HIV acquisition with respect to men or women that do not move outside the rural study community. The solid vertical lines show approximate distances from the rural study community to the metropolitan areas of Richards Bay, Durban, and Johannesburg/Pretoria.

**Fig. 3:**
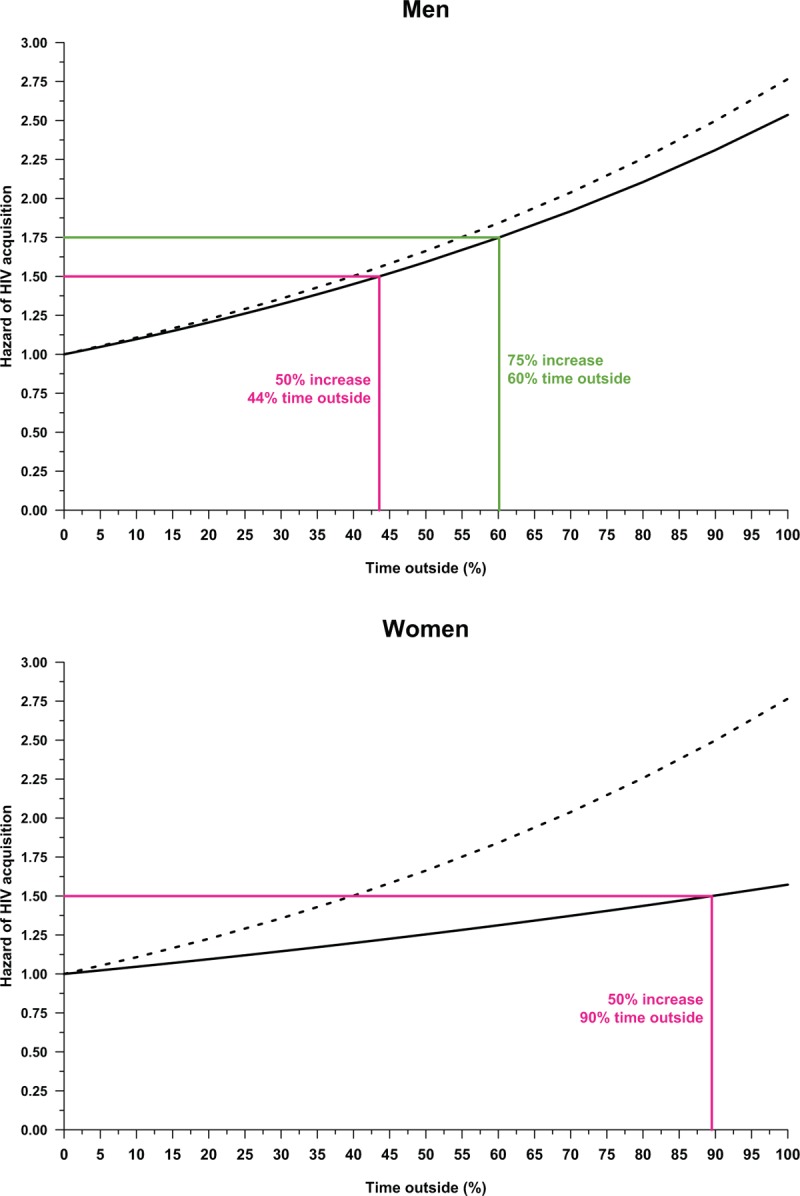
Relationship between the hazard of HIV acquisition and time outside the rural study community expressed in years for men (top) and women (bottom).

In contrast to the distance–HIV risk relationship, the relationship between time spent outside the rural study community and HIV risk is approximately linear (Fig. 3). Every year spent outside the study area conferred substantial additional risk to men and women, but the relationship was more muted for women: adjusted hazard ratio (AHR) = 2.54 (95% CI 1.67–3.85) for men and aHR = 1.57 (95%CI 1.14–2.17) for women (see Table S5 in the Supplemental Material). Men that reside outside the rural study community 44% of the year increased their hazard of acquiring HIV by 50%. To attain this same increase in HIV risk, a woman needed to reside more than 90% of the year outside the rural study community.

The black solid lines give the adjusted hazard of HIV acquisition for time outside, whereas the black dotted lines give the unadjusted hazard of HIV acquisition. The colored lines show the percentage of time spent outside the rural study community that correspond with a 50% or a 75% increase in the adjusted hazard of HIV acquisition with respect to men or women that do not move outside the rural study community.

## Discussion

In this study, we have investigated the causal impact of detailed space-time patterns of human mobility on the risk of subsequent HIV acquisition in a population cohort of 17 743 repeat-testers who were HIV uninfected at baseline. The novelty of our work comes from using the complete geolocated residential histories of every repeat-tester in this cohort that comprises locations inside the rural study community and elsewhere in South Africa. These findings support our key hypothesis that repeat-testers that periodically change their residences over larger distances, or spend larger periods of time outside the rural study community are at a significantly higher risk of acquiring HIV. However, one of the most surprising findings of our work is that even relatively short-distance movements confer substantial additional HIV risk to both men and women. Furthermore, increased spatiotemporal mobility has a stronger link in HIV risk in men: the same levels of risk of HIV acquisition are estimated to occur when men change their residences over shorter average distances per year than women, or when men reside for shorter periods of time outside the rural study community than women.

Larger average migration distances per year and increased periods of residence outside the rural study community represent proxy for key risk factors of HIV acquisition such as increased number of sexual partners, increased likelihood of risky sexual behavior, detachment from family, friends, community, and social norms, increased vulnerability, or lower socioeconomic status. Although some of these HIV acquisition risk factors are self-reported and are therefore hard to measure or confirm, our migration measures can be objectively determined when complete geolocated residential histories are available. As such, the migration indices we proposed objectively quantify individuals’ risks for seroconverting because they are not affected by inherent biases that arise from employing measures that are based on self-reports of sexual histories.

One of the limitations of this study comes from our determination of the geolocations of place of residency outside the rural study community based on descriptions provided by family, friends, or relatives of repeat-testers during face-to-face interviews that occurred while these individuals were absent from the rural study community. These descriptions may not always provide complete geospatial information of the residencies occupied by the repeat-testers. It is possible, for example, that external migrations between two locations outside the rural study community were not captured during the household interviews. In this study, we were unable to consider information on ART uptake in the population under surveillance. For this rural study community, such data are available for only some of the repeat-testers included in our study cohort. The inclusion of these data would thus have led to a decrease in the sample size available. Nevertheless, exploring the relationship among migration, ART uptake and the risk of HIV acquisition is a worthwhile research effort which we are currently undertaking.

This in-depth analysis of a population cohort in a rural sub-Saharan African population has demonstrated the vulnerability of individuals migrating large distances to HIV infection. Over the last decade, a large body of research has shown that mobile individuals typically learn about their HIV status a particularly long time after infection [38]. In addition, migrants face challenges in linking to and remaining in HIV treatment [38]. To the best of our knowledge, such a detailed characterization of the impact of the space-time dimensions of mobility on HIV acquisition has not been established in the existing literature. Our study provides more rigorous criteria for identifying individuals who are at risk of acquiring HIV, and repeatedly move in and out of the Africa Health Research Institute study community. Our methodological approach can be applied to any other HIV surveillance site that collects residential histories. If we want to drive the HIV epidemic to low levels of endemicity and meet the UNAIDS 90–90–90 targets [39], we have to develop novel methods to reach the highly vulnerable population of mobile individuals. There is an urgent need to develop treatment and prevention strategies that cater for the peculiar needs of migrants and their families in hyperendemic sub-Saharan African settings.

## Acknowledgements

T.B. and F.T. received funding from the Wellcome Trust and from the US National Institute of Child Health and Human Development (NICHD R01-HD084233) and National Institute of Allergy and Infectious Diseases (NIAID R01-AI124389). F.T. was additionally supported by South African MRC Flagship (MRC-RFA-UFSP-01–2013/UKZN HIVEPI) grant as well as a UK Academy of Medical Sciences Newton Advanced Fellowship (NA150161). T.B. was additionally supported by the Alexander von Humboldt Professorship Award from the Alexander von Humboldt Foundation, Germany, and by the NIH Fogarty International Center (FIC D43-TW009775).

A.D. and F.T. conceived the study, and performed the data analyses. A.D., T.B., A.V., and F.T. wrote the manuscript.

### Conflicts of interest

There are no conflicts of interest.

## Supplementary Material

Supplemental Digital Content

## Figures and Tables

**Table 1:** Percentage of men and women who migrated outside the rural study community.

	Men		Women	
Sex	At least once (%; 95% CI)	Two or more times (%; 95% CI)	At least once (%; 95% CI)	Two or more times (%; 95% CI)
Age stratum (years)				
15–19	9.66 (8.72–10.59)	2.34 (1.86–2.81)	13.48 (12.44–14.53)	4.64 (4.00–5.28)
20–24	22.90 (21.34–24.46)	11.08 (9.91–12.24)	26.06 (24.57–27.55)	14.83 (13.62–16.03)
25–29	23.30 (20.87–25.73)	10.83 (9.05–12.62)	19.11 (17.15–21.07)	10.33 (8.81–11.84)
30–34	15.50 (12.60–18.40)	6.83 (4.81–8.85)	8.25 (6.56–9.94)	4.72 (3.41–6.02)
35–39	13.97 (10.80–17.15)	7.42 (5.02–9.82)	6.15 (4.70–7.60)	3.03 (1.99–4.06)
40–44	10.17 (7.42–12.93)	5.41 (3.35–7.47)	3.91 (2.87–4.95)	1.58 (0.91–2.25)
≥45	6.33 (5.09–7.56)	4.37 (3.33–5.41)	2.76 (2.26–3.25)	1.87 (1.46–2.28)
Calendar year				
2004	4.46 (3.66–5.27)	0.40 (0.15–0.65)	1.92 (1.48–2.37)	0.03 (0.00–0.08)
2005	6.14 (5.34–6.95)	0.82 (0.52–1.12)	2.52 (2.08–2.97)	0.06 (0.00–0.13)
2006	7.99 (7.10–8.88)	1.46 (1.06–1.85)	1.92 (1.55–2.30)	0.04 (0.00–0.09)
2007	7.72 (6.87–8.57)	1.02 (0.70–1.34)	2.09 (1.74–2.44)	0.06 (0.00–0.12)
2008	8.05 (7.20–8.90)	1.54 (1.16–1.92)	2.70 (2.32–3.09)	0.09 (0.02–0.16)
2009	8.24 (7.36–9.12)	1.32 (0.95–1.68)	3.14 (2.72–3.55)	0.19 (0.09–0.30)
2010	6.97 (6.15–7.80)	1.93 (1.48–2.37)	2.95 (2.55–3.35)	0.35 (0.21–0.49)
2011	5.06 (4.32–5.81)	1.23 (0.85–1.60)	2.60 (2.21–2.99)	0.22 (0.10–0.33)
2012	3.93 (3.20–4.67)	1.41 (0.96–1.86)	1.92 (1.57–2.28)	0.09 (0.01–0.16)
2013	2.77 (2.11–3.44)	0.77 (0.41–1.12)	0.81 (0.56–1.05)	0.00
2014	0.94 (0.43–1.44)	0.14 (0.00–0.34)	0.28 (0.11–0.46)	0.03 (0.00–0.08)

CI, confidence interval.

**Table 2:** Summary of the estimates of adjusted hazard ratios associated with our two migration indices.

	Men		Women	
Risk factor	Adjusted hazard ratio (95% CI)	*P* value	Adjusted hazard ratio (95% CI)	*P* value
Time outside	2.54 (1.67–3.85)	<0.0001	1.57 (1.14–2.17)	0.00602
Log (1 + migration distance)	1.12 (1.06–1.17)	<0.0001	1.09 (1.05–1.13)	<0.0001

We fitted Cox proportional hazards models for men and women men that estimate repeat-testers’ hazard of HIV seroconversion conditional on each migration index, and on several known sexual and sociodemographic risk factors of HIV acquisition: reporting more than one partner in the previous 12 months over the duration of the study, marital status, number of years of education, perceived financial status, age, age^2^, and age^3^. The full output from these models is given in Tables S5 and S6 in the Supporting Information. CI, confidence interval.
