# Supplementary material for: Space-time migration patterns and risk of HIV acquisition in rural South Africa
Source: AIDS. 2016 Dec 2;31(1):137–45. doi: 10.1097/QAD.0000000000001292 (PMC5131684; doi:10.1097/QAD.0000000000001292)
Supplement: Supplemental Digital Content [file aids-31-137-s001.docx]

Supporting Information for ``Space-time migration patterns and risk of HIV acquisition in rural South Africa: a population-based cohort study "

Authors: **Adrian DOBRA^a^, Till BÄRNIGHAUSEN^b^, Alain VANDORMAEL^c^, and Frank TANSER^d^**

^a^Department of Statistics, Department of Biobehavioral Nursing and Health Systems, Center for Statistics and the Social Sciences, and Center for Studies in Demography and Ecology, University of Washington, Seattle, Washington, USA.
Email: [adobra@uw.edu](mailto:adobra@uw.edu)

^b^Africa Health Research Institute, University of KwaZulu-Natal, Mtubatuba, South Africa, Institute for Public Health, Faculty of Medicine, University of Heidelberg, Heidelberg, Germany, and Department of Global Health and Population, Harvard T.H. Chan School of Public Health, Boston, Massachusetts, USA.
Email: [tbaernig@hsph.harvard.edu](mailto:tbaernig@hsph.harvard.edu)

^c^Africa Health Research Institute, University of KwaZulu-Natal, Mtubatuba, South Africa.
Email: [vando026@umn.edu](mailto:vando026@umn.edu)

^d^Africa Health Research Institute, and School of Nursing and Public Health, University of KwaZulu-Natal, Durban, South Africa.

Centre for the AIDS Programme of Research in South Africa – CAPRISA, University of KwaZulu-Natal, Congella, South Africa.
Email: [ftanser@gmail.com](mailto:ftanser@gmail.com)

## S1. Systematic review of recent literature linking migration and HIV

**Table S1. Review of recent papers linking mobility and HIV infection**

| Authors | Aims | Results |
| --- | --- | --- |
| Bärnighausen et al. ^[1]^ | Longitudinal cohort study in rural South Africa (KwaZulu-Natal) investigating socioeconomic factors of HIV spread. | After controlling for sex, age, education, wealth, household expenditures and place of residence, the hazard of acquiring HIV is almost double in migrants vs. non-migrants. |
| Camlin et al. ^[2]^ | Examines the relationships between migration and HIV infection for women and men in rural South Africa (KwaZulu-Natal). | Each additional partner increased odds of HIV infection by 46% for female migrants which indicates a substantially higher risk compared to female non-migrants (22%), male non-migrants (3%), and male migrants (non-significant). |
| Deane, Parkhurst and Johnston ^[3]^ | Discusses the occasionally contradictory published results on the complex relationships between migration and HIV infection risk factors, together with a key example from an epidemiological cohort from Kisesa, Tanzania. | The various definitions of mobile and non-mobile groups employed in the literature influence the conclusions of studies linking HIV acquisition with risky sexual behavior and migration. Improved approaches should account for reasons for migration, who migrates, and key epidemiological features of origin and destination areas. |
| Saggurti et al. ^[4]^ | Cross-sectional risky sexual behavioral study of non-migrants, returned migrants and active migrants in two Indian districts with high levels of male out-migration. | Returned and active migrants have increased risky sexual behavior (paid and unprotected se9x) compared to non-migrants. Migrants maintain their risky sexual behavior in the places of origin and the places of destination. |
| Vissers et al. ^[5]^ | Extended a model called STDSIM for simulating the natural history and transmission of HIV and other STDs to also simulate mobility patterns in Tanzania. | Although migrants represent a relatively small part of a population, the effectiveness of interventions (e.g. condom promotion or health education) in reducing HIV transmission could be substantially reduced if migrants are not reached. |
| Anglewicz ^[6]^ | This study employs a longitudinal data from rural Malawi to assess the relationship between migration and HIV. | While this study argues that migrants are more likely to be HIV positive, it also finds evidence that HIV positive individuals are more likely to migrate. Thus, HIV acquisition could precede rather than follow migration. |
| Goldenberg et al. ^[7]^ | Review of 275 papers and 22 studies focusing on the link between migration and HIV in Central America and Mexico. | Migrants are more likely to engage in risky sexual behavior. In the same time, the likelihood of HIV acquisition can increase due to social isolation, gender inequalities, stigma and discrimination of migrants. |
| Veary ^[8]^ | This paper reviews empirical studies linking migration into urban areas and health. | Individuals who migrate from rural to urban areas are more likely to be healthy. However, as migrants are unlikely to properly integrate in the health care system, they become sick and return to their natal rural areas for palliative care. The economic benefits of rural areas from migration to urban areas are often offset by the burden to care for migrants that are too sick to work and return home. |
| Weine and Kashuba ^[9]^ | Review of 97 papers focusing on risk factors of HIV acquisition in labor migrants in Africa, the Americas, Europe, South East Asia and Western Pacific. | The study reveals key determinants associated with HIV risk in the levels of policy (3), sociocultural (3), sexual practices (4), and health and mental health (5), together with the size of their effects. |
| Cassels et al. ^[10]^ | This paper investigates the relationship between short-term mobility and partnership concurrency among men using the 2010-11 Zimbabwe Demographic and Health Survey. | Men who traveled more had an increased likelihood to have concurrent relationships compared with men that did not travel. Thus short-term travel is an important risk factor in HIV transmission as it enables concurrent relationships. |
| Camlin et al. ^[11]^ | This paper presents the underlying social contexts and processes that enable HIV transmission and acquisition among female migrants in Kisumu, Kenya. | Women often migrate due to changes in marital status and abusive relationships. After reaching their destinations, women face significant pressure to take multiple sexual partners, and possibly engage in transactional sex. As such, migrant women have a high risk of HIV transmission and acquisition both at origin and at the destination of their travel. |
| Camlin, Snow and Hosegood ^[12]^ | Examines differential patterns of migration among men and women in rural South Africa (KwaZulu-Natal). | Both men and women out-migrated to seek work. Women tend to in-migrate, while men tend to out-migrate. Migration rates in women are slightly higher than men’s. |
| Cassels et al. ^[13]^ | One-year retrospective study in Agbogbloshie, Ghana that collected retrospective data on sexual partners, migration, short-term mobility and HIV infection. | High levels of short-term mobility were recorded: residents averaged 7.3 overnight trips in the last year, while 37% of men and 9% of women had multiple sexual partners in the last year. |
| Cassels, Jenness and Khanna ^[14]^ | This paper introduces a conceptual framework for representing the effects of mobility as a driver for risky sexual behavior, for enabling spatiotemporal links between sub-epidemics, and for characterizing the displacement effect of sending/receiving communities. | Treatment as prevention has shifted the focus of migration patterns with respect to HIV: migrants must be the target of prevention efforts to improve linkage to care and ART adherence. |
| Kenyon et al. ^[15]^ | Using ecological level data from 141 countries, this study investigates the association between migration intensity and peak national HIV prevalence. | When evaluated at the population level, there does not seem to exist any significant relationship between migration and HIV prevalence. |
| Lurie and Williams ^[16]^ | This paper discusses the role of migration in the transmission and dissemination of tuberculosis and HIV in South African rural areas. | Young men who migrated to seek work and subsequently returned to their origin rural areas acted as key drivers of two major epidemics nearly 100 years apart. The authors discuss the emerging role of disease-induced migration vs. migration-induced diseases. The former is crucial for continued access to treatment and care. |
| Rai et al. ^[17]^ | This is a behavioral study of 639 male circular migrants from Azamgarh district, India focusing on the role of circular migrants as a bridge population between areas with high and low HIV prevalence. | Groups of circular migrants vary in size and sexual behavior across locations and time. As a result of their behavioral heterogeneity, only 1 in 5 circular migrants seem to connect sexual transmission networks from geographically separated high and low HIV prevalence areas. |
| McGrath et al. ^[18]^ | This is a longitudinal behavioral study in rural South Africa (KwaZulu-Natal) focusing on the associations between sexual behavior, migration and HIV. | Current residents of the surveillance area with a recent history of migration had an increased likelihood of being seropositive compared to residents who did not migrate. |
| Thapa et al. ^[19]^ | This paper investigates risk factors for HIV acquisition among spouses of male labor migrants in Nepal. | Literacy status of the woman was the only social factor associated with HIV infection. However, the men-related factors included, among others, country of migration and age of at first migration episode. |
| Palk and Blower ^[20]^ | This study employed georeferenced Demographic and Health Survey data from Lesotho to construct country-level maps of mobility stratified by gender. | Men who traveled often (21% of the surveyed individuals) had an increased likelihood of HIV infection than men that did not travel. However, men who traveled less often and women that traveled did not have an increased likelihood of HIV infection. |

Notes: These papers have been published between 2007 and 2015.

## S2. Geocoding Places of Residency Outside the Surveillance Area

The raw descriptions of places of residency that were not associated with a bounded structure inside the study area were cleaned by removing special characters, words without precise meaning (e.g., “don’t know”, “missing”, “non-applicable”, “default”, “refused”, “query”, “elsewhere”, “foreign country”, “outside sub-Saharan Africa”), and by correcting spelling mistakes. These corrections have been initially carried out using computer code, followed by manual checks. When ambiguities in the resulting descriptions still seem to exist, string matching techniques were applied to identify the closest descriptions without ambiguities. Decisions about replacing a description of a place of residency with its closest match have been taken by a trained research assistant. Every description of place was labeled as “inside South Africa” or “outside South Africa”.

The cleaned versions of the description of places labeled “inside South Africa” have been geocoded using three geocoding services: HERE Geocoder API (http://dev.here.com/mapsAPI_geocoder/), Bing Maps REST Services API (<http://msdn.microsoft.com/en-us/library/ff701715.aspx>) and Google Maps Geocoding API (<https://developers.google.com/maps/documentation/geocoding/intro>). These three services allow the determination of latitude and longitude coordinates of a location specified by a text address. The text address can be incomplete or spelled incorrectly. These three geocoding APIs take a text address, match it against their internal databases of known addresses and return latitude and longitude coordinates if any satisfactory match has been identified. When no satisfactory match has been identified, the APIs do not return any coordinates. The use of three geocoding services provides robustness of the coordinates identified because HERE, Bing and Google employ various string matching rules and techniques. The internal databases of known addresses of these services could also have various degrees of coverage and quality for different parts of South Africa.

The HERE, Bing and Google APIs were employed with queries of the form “XXX, South Africa” where “XXX” is a cleaned description of a place of residency labeled “inside South Africa”. A total of 10,155 queries associated with each unique description of a place of residency were run. Those queries that were considered too ambiguous to yield a valid geolocation were dropped. HERE returned the latitude and longitude coordinates associated with 5,366 queries (52.84%). Bing returned the latitude and longitude coordinates associated with 3,990 queries (39.29%). Google returned the latitude and longitude coordinates associated with 5,993 queries (59.02%). The latitude and longitude coordinates of 2,587 queries (25.48%) were identified by all three APIs. The coordinates of 1,347 queries (13.26%) were identified by HERE and Google APIs but not by the Bing API. The coordinates of 787 queries (7.75%) were identified by the Bing and Google APIs but not by the HERE API. The coordinates of 329 queries (3.24%) were identified by the Bing and HERE APIs but not by the Google API. The coordinates of 287 queries (2.82%) were identified only by the Bing API. The coordinates of 1,103 queries (10.86%) were identified only by the HERE API. The coordinates of 1,212 queries (11.94%) were identified only by the Google API. Finally, the coordinates of 2,503 queries (24.65%) were not identified by any of the three APIs. When two or all there of the geocoding APIs successfully geocoded a query, the coordinates they returned were quite consistent: the corresponding distances between them were less than 5 km. The exceptions were manually checked. The latitude and longitude coordinates that were closest to the study area were chosen for the queries for which multiple latitude and longitude coordinates were available. The queries that were successfully geo-located by a single API were manually checked. The decision whether to use the latitude and longitude coordinates was taken on a case by case basis after assessing the validity of the original query.

**S3. Additional explanations related to the construction of migration indices**

We create time-varying measures of migration that express the dynamic spatial and temporal extent of migration patterns recorded for repeat-testers. Longitudinally recorded migration episodes capture heterogeneous patterns of movement of repeat-testers that repeatedly leave the study area, come back to the study area, possibly move between residences inside the study area, then move out again. Some repeat-testers move between two residencies outside the study area without moving back into the study area. Other repeat-testers return to the same bounded structure inside the study area after their external migration episodes. Yet, some repeat-testers return from an external migration episode to a bounded structure inside the study area that is different from the bounded structure they resided in when they outmigrated. Some repeat-testers internally migrate, but might never reside outside the study area.

Our first migration index which we call *time outside* represents the proportion of time per year spent outside the study area. It is exclusively a temporal measure that is independent of the distance between consecutive residences occupied by a repeat-tester. For example, time outside cannot differentiate between two hypothetical repeat-testers A and B that change their residences in and out of the study area at the exact same dates, but reside in Johannesburg and in Richards Bay, respectively, when living outside the study area. Because Johannesburg is located farther away from the study area than Richards Bay, the expenses and time needed to visit the study area for A are significantly larger than for B. As such, A will likely visit the study area less than B, and consequently their degree of separation from their sexual partners, family and friends that reside in the study area is likely to be larger for A compared to B. For this reason, we introduce five additional time-varying measures of migration that depend on the distances between consecutive residencies occupied by a repeat-tester, and also on the time elapsed between these residency changes.

Our second migration index is called *migration distance*. It is calculated as the ratio between the sum of distances between consecutive residences occupied during episodes that coincide or precede a reference episode, and the sum of the durations of all the exposure episodes that coincide or precede the same reference episode. Migration distance takes into account distances between any two consecutive residences occupied by a repeat-tester irrespective of their location outside or inside the study area. While the migration patterns of the two hypothetical repeat-testers A and B record the same values for the time outside measure because they move in and out the study area in the same time, they give larger values of migration distance for A than for B because A moves over a larger distance than B.

Migration distance captures external migration episode as well as internal migration episodes. For repeat-testers, time outside can be interpreted as the average percentage of the year spent outside the study area in contiguous time intervals defined by sequential exposure episodes since the start of their exposure. Our distance-dependent measure can be interpreted as the average distance per year a repeat-tester has migrated in the same contiguous time intervals defined by sequential exposure episodes.

**Table S2. Descriptive statistics of the times between the date of the last HIV negative test and the date of the first HIV positive test for men (N=547, 7.82%) that seroconverted**

| Age stratum (years) | N (%) | Min | 2.5% | Median (IQR) | Mean | 97.5% | Max |
| --- | --- | --- | --- | --- | --- | --- | --- |
| 15-19 | 147  (26.87%) | 0.73 | 0.87 | 3.71 (4.71) | 4.34 | 9.99 | 11.00 |
| 20-24 | 191 (34.92%) | 0.61 | 0.91 | 2.87 (3.77) | 3.66 | 9.39 | 10.83 |
| 25-29 | 78 (14.26%) | 0.34 | 0.69 | 2.13 (2.14) | 2.93 | 8.66 | 9.47 |
| 30-34 | 38  (6.95%) | 0.83 | 0.91 | 2.59 (3.24) | 3.32 | 8.50 | 10.05 |
| 35-39 | 30  (5.48%) | 0.97 | 0.98 | 2.09 (3.50) | 3.40 | 8.75 | 10.06 |
| 40-44 | 22 (4.02%) | 1.02 | 1.05 | 3.06 (2.97) | 3.29 | 6.25 | 6.56 |
| ≥45 | 41 (7.50%) | 0.73 | 0.94 | 2.50 (2.70) | 2.74 | 6.25 | 7.02 |
| All ages | 547 (100%) | 0.34 | 0.88 | 2.86 (3.48) | 3.62 | 9.47 | 11.00 |

Notes: Time is expressed in years.

**Table S3. Descriptive statistics of the times between the date of the last HIV negative test and the date of the first HIV positive test for women (N=1478, 13.75%) that seroconverted**

| Age stratum (years) | N (%) | Min | 2.5% | Median (IQR) | Mean | 97.5% | Max |
| --- | --- | --- | --- | --- | --- | --- | --- |
| 15-19 | 607 (41.07%) | 0.44 | 0.91 | 2.41 (2.75) | 3.08 | 8.75 | 11.04 |
| 20-24 | 429 (29.03%) | 0.35 | 0.77 | 2.06 (2.61) | 2.80 | 8.50 | 10.78 |
| 25-29 | 149 (10.08%) | 0.61 | 0.81 | 2.06 (2.89) | 2.91 | 8.44 | 10.50 |
| 30-34 | 81 (5.48%) | 0.70 | 0.91 | 1.96 (3.01) | 2.75 | 8.94 | 10.06 |
| 35-39 | 49 (3.32%) | 0.80 | 0.93 | 2.32 (2.90) | 3.22 | 8.32 | 8.37 |
| 40-44 | 69 (4.67%) | 0.27 | 0.82 | 2.07 (3.02) | 2.89 | 8.41 | 9.95 |
| ≥45 | 94 (6.35%) | 0.68 | 0.87 | 1.96 (2.16) | 2.55 | 8.21 | 9.55 |
| All ages | 1478 (100%) | 0.27 | 0.87 | 2.12 (2.82) | 2.93 | 8.64 | 11.04 |

Notes: Time is expressed in years.

**Table S4. Crude HIV incidence rates**

| Gender |  | Person-years | HIV sero-conversions (#) | Crude HIV incidence rate per 100 person-years of observation  (95% CI) |
| --- | --- | --- | --- | --- |
| Men |  | | | |
|  | **Age stratum (years)** | | | |
|  | 15-19 | 7908 | 72 | 0.91 (0.70-1.12) |
|  | 20-24 | 5504 | 203 | 3.69 (3.19-4.19) |
|  | 25-29 | 2088 | 121 | 5.79 (4.79-6.80) |
|  | 30-34 | 1142 | 44 | 3.85 (2.74-4.97) |
|  | 35-39 | 951 | 34 | 3.57 (2.39-4.75) |
|  | 40-44 | 1100 | 30 | 2.72 (1.76-3.69) |
|  | ≥45 | 6589 | 43 | 0.65 (0.46-0.85) |
|  | **Calendar year** | | | |
|  | 2004 | 1618 | 35 | 2.16 (1.45-2.87) |
|  | 2005 | 2416 | 25 | 1.03 (0.63-1.44) |
|  | 2006 | 2665 | 67 | 2.51 (1.92-3.11) |
|  | 2007 | 2877 | 73 | 2.54 (1.96-3.11) |
|  | 2008 | 3114 | 82 | 2.63 (2.07-3.20) |
|  | 2009 | 2963 | 84 | 2.83 (2.24-3.43) |
|  | 2010 | 2815 | 57 | 2.02 (1.50-2.54) |
|  | 2011 | 2458 | 53 | 2.16 (1.58-2.73) |
|  | 2012 | 2121 | 45 | 2.12 (1.51-2.73) |
|  | 2013 | 1603 | 24 | 1.50 (0.90-2.09) |
|  | 2014 | 629 | 2 | 0.32 (0-0.76) |
| Women |  |  |  |  |
|  | **Age stratum (years)** | | | |
|  | 15-19 | 7604 | 388 | 5.10 (4.61-5.60) |
|  | 20-24 | 6257 | 570 | 9.11 (8.40-9.82) |
|  | 25-29 | 2732 | 192 | 7.03 (6.07-7.99) |
|  | 30-34 | 2106 | 91 | 4.32 (3.45-5.19) |
|  | 35-39 | 2634 | 56 | 2.13 (1.57-2.68) |
|  | 40-44 | 3407 | 62 | 1.82 (1.37-2.27) |
|  | ≥45 | 20367 | 117 | 0.57 (0.47-0.68) |
|  | **Calendar year** | | | |
|  | 2004 | 2194 | 81 | 3.69 (2.90-4.48) |
|  | 2005 | 3267 | 135 | 4.13 (3.45-4.81) |
|  | 2006 | 3664 | 166 | 4.53 (3.86-5.20) |
|  | 2007 | 4412 | 181 | 4.10 (3.52-4.69) |
|  | 2008 | 5309 | 209 | 3.94 (3.41-4.46) |
|  | 2009 | 5393 | 164 | 3.04 (2.58-3.50) |
|  | 2010 | 5372 | 154 | 2.87 (2.42-3.31) |
|  | 2011 | 5127 | 150 | 2.93 (2.46-3.39) |
|  | 2012 | 4770 | 144 | 3.02 (2.53-3.50) |
|  | 2013 | 3931 | 67 | 1.70 (1.30-2.11) |
|  | 2014 | 1667 | 25 | 1.50 (0.92-2.08) |

Notes: In men (N=6,995, upper panel) and women (N=10,748, lower panel), by age stratum and calendar year, 2004 – 2014. Person-years are based on midpoint imputation of the seroconversion date between the date of the last negative and first positive test for HIV sero-converters, and on the date of the last negative test for those who are censored. CI = confidence interval.

**Figure S1. Relationship between the duration (expressed in years) of periods of residence outside the rural study community and distances (expressed in km) migrated in men**

*
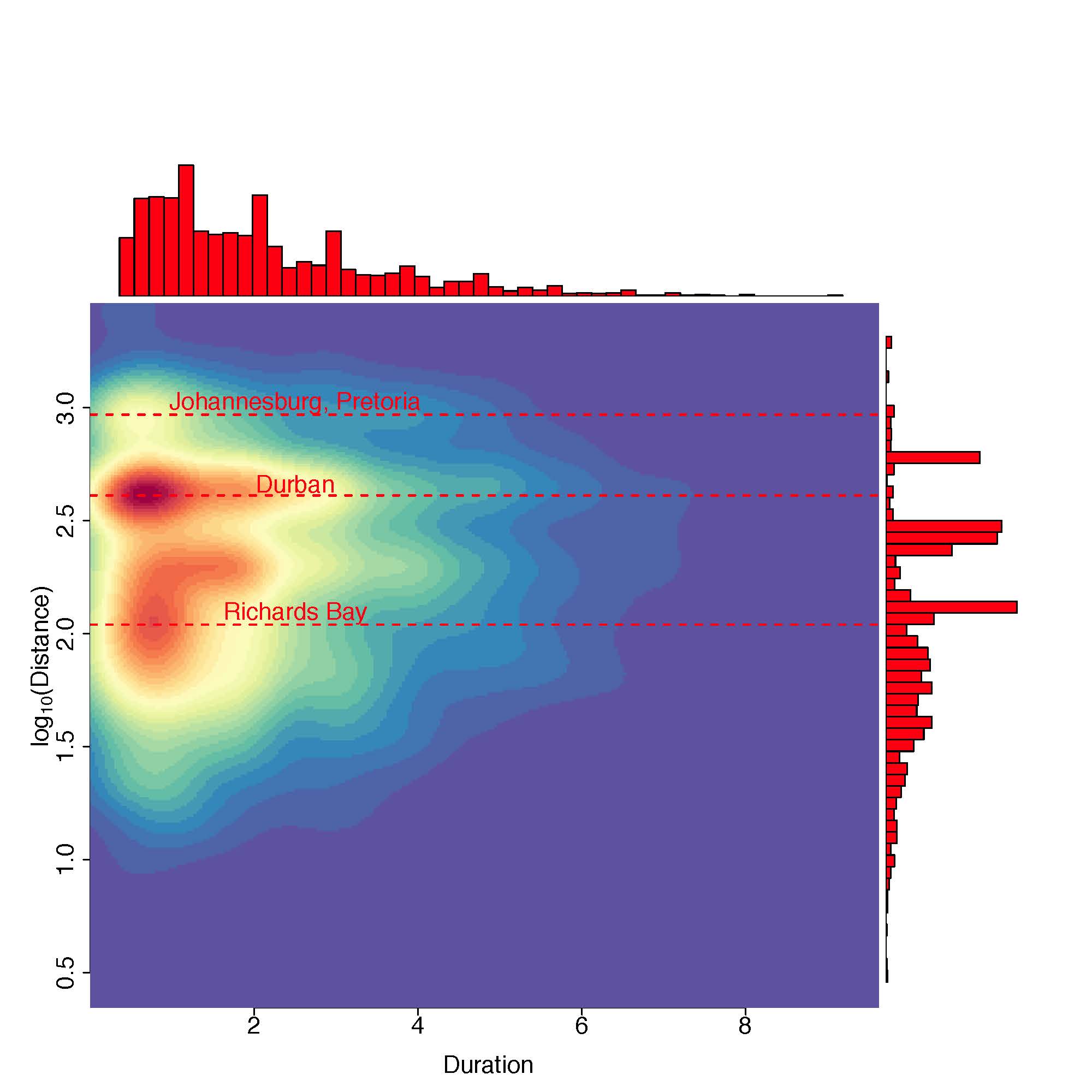
*

Notes: For each man, we determined his periods of residence outside the rural study community and calculated the sum of the distances between the locations of consecutive residences he occupied. Colors closer to red indicate higher densities of (duration, distance) points, while colors closer to blue indicate smaller densities. The plot shows two well defined clusters of migrations outside the rural study community that last up to approximately one year, and involve travel to destinations in the KwaZulu-Natal province, most often to two cities, Richards Bay and Durban. There are also two smaller clusters, one that involves travel to more distant destinations in Johannesburg and Pretoria in the Gauteng province, and another cluster that involves travel to destinations in the KwaZulu-Natal province with much longer durations of residence outside the rural study community between two and three years. The horizontal lines show the length of round trips to Richards Bay, Durban, Johannesburg/Pretoria. These trips represent the equivalent of an individual who moves from the rural study community to one of these cities, then moves back to the rural study community.

**Figure S2. Relationship between the duration (expressed in years) of periods of residence outside the rural study community and distances (expressed in km) migrated in women**

**
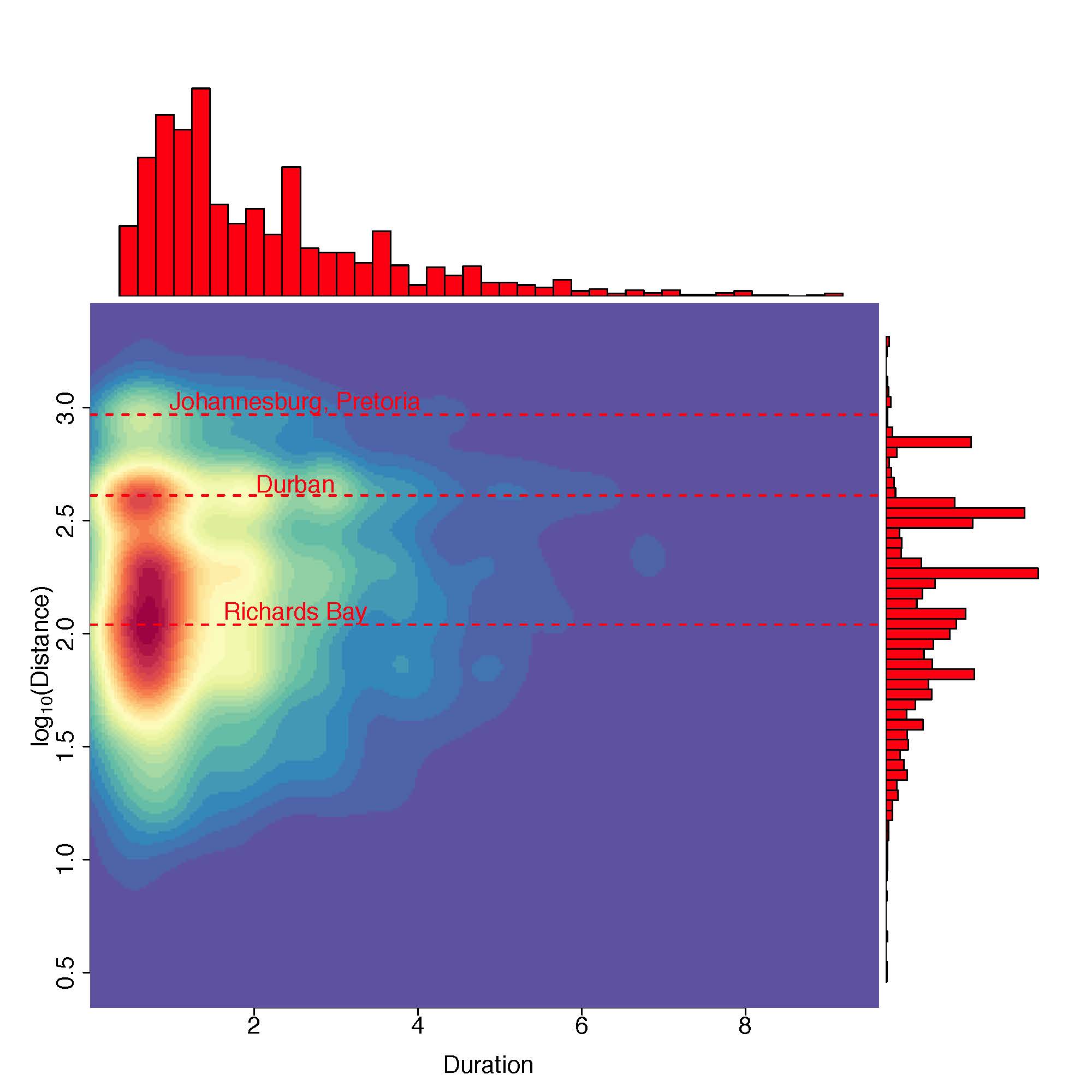
**

Notes: For each woman, we determined her periods of residence outside the rural study community and calculated the sum of the distances between the locations of consecutive residences she occupied. Colors closer to red indicate higher densities of (duration, distance) points, while colors closer to blue indicate smaller densities. The clusters of outside the rural study community migrations of women that are visible in this plot are quite similar to the clusters of outside the rural study community migrations for men – see Figure S1. The horizontal lines show the length of round trips to Richards Bay, Durban, Johannesburg/Pretoria. These trips represent the equivalent of an individual who moves from the rural study community to one of these cities, then moves back to the rural study community.

**Table S5. Full output from Cox proportional hazards models for men and women that estimate repeat-testers’ hazard of HIV seroconversion conditional on time outside, and on sexual and sociodemographic factors**

|  | | Men | | Women | |
| --- | --- | --- | --- | --- | --- |
| Risk Factor | | **Adjusted hazard ratio (95% CI)** | **p value** | **Adjusted hazard ratio (95% CI)** | **p value** |
| Time Outside | | 2.54 (1.67-3.85) | <0.0001 | 1.57 (1.14-2.17) | 0.00602 |
| Sexual Partners | |  |  |  |  |
|  | One or less (baseline) | 1.00 |  | 1.00 |  |
|  | Two or more | 1.62 (1.35-1.93) | <0.0001 | 1.91 (1.51-2.42) | <0.0001 |
| Marital Status | |  |  |  |  |
|  | Single (baseline) | 1.00 |  | 1.00 |  |
|  | Married, monogamous | 0.39 (0.24-0.62) | <0.0001 | 0.51 (0.41-0.63) | <0.0001 |
|  | Married, polygamous | 1.40 (0.44-4.47) | 0.56464 | 0.51 (0.41-0.63) | 0.01161 |
| Years of education | | 1.00 (0.97-1.03) | 0.85978 | 1.00 (0.98-1.02) | 0.72179 |
| Perceived financial status | |  |  |  |  |
|  | Unknown (baseline) | 1.00 |  | 1.00 |  |
|  | Comfortable or just getting by | 1.79 (1.15-2.78) | 0.00964 | 1.79 (1.37-2.36) | <0.0001 |
|  | Poor | 1.96 (1.36-2.82) | 0.00028 | 1.76 (1.38-2.24) | <0.0001 |
| Age | | 0.97 (0.95-0.98) | <0.0001 | 0.92 (0.91-0.93) | <0.0001 |
| Age^2^ | | 1.00 (0.99-1.00) | <0.0001 | 1.00 (1.00-1.00) | <0.0001 |
| Age^3^ | | 1.00 (1.00-1.00) | <0.0001 | 1.00 (1.00-1.00) | <0.0001 |

Notes: All the risk factors with the exception of sexual partners are time dependent.

Table S6. Full output from Cox proportional hazards models for men and women that estimate repeat-testers’ hazard of HIV seroconversion conditional on migration distance, and on sexual and sociodemographic factors

|  | | Men | | Women | |
| --- | --- | --- | --- | --- | --- |
| Risk factor | | **Adjusted hazard ratio (95% CI)** | **p value** | **Adjusted hazard ratio (95% CI)** | **p value** |
| log(1+Migration Distance) | | 1.12 (1.06-1.17) | <0.0001 | 1.09 (1.05-1.13) | <0.0001 |
| Sexual Partners | |  |  |  |  |
|  | One or less (baseline) | 1.00 |  | 1.00 |  |
|  | Two or more | 1.60 (1.34-1.92) | <0.0001 | 1.89 (1.50-2.40) | <0.0001 |
| Marital Status | |  |  |  |  |
|  | Single (baseline) | 1.00 |  | 1.00 |  |
|  | Married, monogamous | 0.39 (0.25-0.62) | <0.0001 | 0.51 (0.41-0.64) | <0.0001 |
|  | Married, polygamous | 1.34 (0.42-4.24) | 0.62337 | 0.52 (0.31-0.87) | 0.01215 |
| Years of education | | 1.00 (0.97-1.03) | 0.89607 | 0.99 (0.97-1.02) | 0.61961 |
| Perceived financial status | |  |  |  |  |
|  | Unknown (baseline) | 1.00 |  | 1.00 |  |
|  | Comfortable or just getting by | 1.41 (0.95-2.09) | 0.08592 | 1.79 (1.39-2.29) | <0.0001 |
|  | Poor | 1.57 (1.16-2.13) | 0.00384 | 1.77 (1.43-2.18) | <0.0001 |
| Age | | 0.97 (0.95-0.98) | <0.0001 | 0.92 (0.91-0.93) | <0.0001 |
| Age^2^ | | 1.00 (0.99-1.00) | <0.0001 | 1.00 (0.99-1.00) | <0.0001 |
| Age^3^ | | 1.00 (1.00-1.00) | <0.0001 | 1.00 (1.00-1.00) | <0.0001 |

Notes: All the risk factors with the exception of sexual partners are time dependent.

## References

1. Bärnighausen T, Hosegood V, Timaeus IM, Newell M-L. **The socioeconomic determinants of HIV incidence: evidence from a longitudinal, population-based study in rural South Africa**. *AIDS* 2007; 21(Suppl 7):S29--S38.

2. Camlin CS, Hosegood V, Newell M-L, McGrath N, Bärnighausen T, Snow RC. **Gender, Migration and HIV in Rural KwaZulu-Natal, South Africa**. *PLoS ONE* 2010; 5(7):e11539.

3. Deane KD, Parkhurst JO, Johnston D. **Linking migration, mobility and HIV**. *Tropical Medicine and International Health* 2010; 15(12):1458–1463.

4. Saggurti N, Mahapatra B, Swain SN, Jain AK. **Male migration and risky sexual behavior in rural India: is the place of origin critical for HIV prevention programs?** *BMC Public Health* 2011; 11(S6).

5. Vissers DCJ, de Vlas SJ, Bakker R, Urassa M, Voeten HACM, Habbema JDF. **The impact of mobility on HIV control: a modelling study**. *Epidemiology and Infection* 2011; 139(12):1845-1853.

6. Anglewicz P. **Migration, Marital Change, and HIV Infection in Malawi**. *Demography* 2012; 49(1):239-265.

7. Goldenberg SM, Strathdee SA, Perez-Rosales MD, Sued O. **Mobility and HIV in Central America and Mexico: a critical review**. *Journal of Immigrant and Minority Health* 2012; 14(1):48-64.

8. Veary J. **Learning from HIV: Exploring migration and health in South Africa**. *Global Public Health* 2012; 7(1):58-70.

9. Weine SM, Kashuba AB. **Labor Migration and HIV Risk: A Systematic Review of the Literature**. *AIDS and Behavior* 2012; 16:1605–1621.

10. Cassels S, Manhart L, Jenness SM, Morris M. **Short-term Mobility and Increased Partnership Concurrency among Men in Zimbabwe**. *PLoS ONE* 2013; 8(6):e66342.

11. Camlin CS, Kwena ZA, Dworkin SL, Cohen CR, Bukusi EA. **“She mixes her business”: HIV transmission and acquisition risks among female migrants in western Kenya**. *Social Science and Medicine* 2014; 102:146-156.

12. Camlin CS, Snow RC, Hosegood V. **Gendered Patterns of Migration in Rural South Africa**. *Population, Space and Place* 2014; 20(6):528-551.

13. Cassels S, Jenness SM, Biney AAE, Ampofo WK, Dodoo FN-A. **Migration, sexual networks, and HIV in Agbogbloshie, Ghana**. *Demographic Research* 2014; 31:861 - 888.

14. Cassels S, Jenness SM, Khanna AS. **Conceptual Framework and Research Methods for Migration and HIV Transmission Dynamics**. *AIDS and Behavior* 2014; 18(12):2302–2313.

15. Kenyon C, Colebunders R, Voeten H, Lurie M. **Migration intensity has no effect on peak HIV prevalence: an ecological study**. *BMC Infectious Diseases* 2014; 14:350.

16. Lurie MN, Williams BG. **Migration and health in Southern Africa: 100 years and still circulating**. *Health Psychology and Behavioral Medicine: An Open Access Journal* 2014; 2(1):34-40.

17. Rai T, Lambert HS, Borquez AB, Saggurti N, Mahapatra B, Ward H. **Circular Labor Migration and HIV in India: Exploring Heterogeneity in Bridge Populations Connecting Areas of High and Low HIV Infection Prevalence**. *Journal of Infectious Diseases* 2014; 210(Suppl 2):S556-S561.

18. McGrath N, Eaton JW, Newell M-L, Hosegood V. **Migration, sexual behaviour, and HIV risk: a general population cohort in rural South Africa**. *The Lancet HIV* 2015; 2(6):e252 - e259.

19. Thapa S, Bista N, Timilsina S, Buntinx F, Mathei C. **Social and behavioural risk factors for HIV infection among the wives of labour migrants in Nepal**. *International Journal of STD & AIDS* 2014; 25(11):793-799.

20. Palk L, Blower S. **Mobility and Circular Migration in Lesotho: Implications for Transmission, Treatment, and Control of a Severe HIV Epidemic**. *Journal of Acquired Immune Deficiency Syndromes* 2015; 68(5):604-608.
